# Artificial intelligence applications in exercise and physical activity for public health: a scoping review

**DOI:** 10.3389/fpubh.2026.1888805

**Published:** 2026-10-09

**Authors:** Sinem Parlakyıldız, Funda Koçak

**Affiliations:** 1 Nevşehir Hacı Bektaş Veli University, Nevşehir, Türkiye; 2 Ankara University, Ankara, Türkiye

**Keywords:** artificial intelligence, digital health, exercise, physical activity, public health

## Abstract

**Background:**

Physical activity and exercise are important components of public health strategies for preventing and managing chronic conditions and promoting health and quality of life. Advances in artificial intelligence (AI) have broadened its applications in this field, from personalized interventions and monitoring to risk prediction, professional decision support, and planning. This scoping review aimed to map the literature on AI-based exercise and physical activity applications from a public health perspective, characterize their functional roles, and examine the scope and maturity of the research.

**Methods:**

This scoping review followed the PRISMA Extension for Scoping Reviews (PRISMA-ScR) and JBI methodological guidance. Web of Science, Scopus, and PubMed were searched for eligible studies published between 2015 and 2025. Two Reviewers independently conducted title and abstract screening and full-text assessment, resolving disagreements by consensus. Of 612 records identified, 601 remained after duplicate removal and 24 studies met the eligibility criteria. The included studies were characterized by publication year, country, study design, AI technology and function, and stage of evidence development. Key findings were synthesized across the identified functional categories.

**Results:**

Among the included studies, publication activity was highest in 2024, while China accounted for the largest number of studies. Five functional categories were identified: Intervention Delivery or Adaptation, Monitoring or Assessment, Prediction or Risk Stratification, Professional Decision Support, and System and Environmental Planning. Research maturity differed across these functions. Technical development and validation studies often reported promising performance, whereas intervention findings were less consistent and did not always show additional benefit over comparison conditions. Many studies remained at the model-development, technical-validation, feasibility, or pilot stage

**Conclusion:**

AI is being used across several functions in exercise and physical activity, although evidence of consistent clinical or public health benefit remains limited. Current findings support its complementary use in personalization, monitoring, prediction, and professional decision support; the available findings do not justify autonomous clinical decision-making. Further controlled studies, external validation, longer follow-up, and real-world evaluation are needed to establish whether technical capabilities translate into sustained physical activity and meaningful health outcomes. Implementation should also account for professional oversight, data protection, and the needs of diverse populations.

## Introduction

Physical activity and exercise are known to support public health (1, 2). Artificial intelligence (AI) continues to evolve rapidly, with expanding applications and potential in the health sector (3–5). AI may contribute to public health by providing opportunities such as creating exercise prescriptions tailored to individuals' specific physical and health conditions and monitoring health status (6). Meeting recommended physical activity levels is an important health indicator, with positive effects on mental wellbeing (7), cardiovascular health (8), and the prevention and management of many disorders, such as diabetes (9). Although activity amount recommendations are determined with specific durations and intensities according to age group (50), customized programs that account for individuals' health status may have a greater impact on the achievement of health goals (10). There are currently numerous physical activity applications offering personalized programs in the AI technology market (11). Beyond exercise prescription, AI-supported applications have been developed for monitoring, behavioral support, personalized guidance, intervention delivery, and the prediction of health-related outcomes (12–14). The studies identified in the present review further include professional decision support and system- and environment-level applications. Classifying these applications according to the function performed by AI provides a basis for organizing the evidence beyond technology or intervention type alone.

Public health is distinguished from clinical medicine by its population-level focus, whereas clinical medicine primarily addresses the diagnosis and treatment of individual patients. Promoting health and preventing disability and both communicable and non-communicable diseases is a collective effort that involves community surveillance, regulation of health determinants, and provision of basic health services (15). International and regional public health agencies have underscored the importance of digital technologies in public health by establishing strategic frameworks designed to harness their potential for improving public health outcomes (16). The last two decades have been characterized by the rapid spread and implementation of digital technologies in public health fields. A wide range of digital technologies, such as mobile applications, social media applications, wearable devices, AI, and big data, have been introduced with the promise of increasing the speed, efficiency, and cost-effectiveness of public health services (17, 18).

A previous scoping review of AI in physical activity examined the use of machine learning, deep learning, and reinforcement learning in physical activity interventions. An et al. (12) identified applications involving the recognition of physical activity behavior patterns, prediction of intervention outcomes, and development of personalized interventions. AI-supported exercise and physical activity applications can also be examined through their broader public health roles, extending from intervention delivery and adaptation to monitoring, risk prediction, professional decision support, and system- or environment-level planning. Examining these functions together offers a different perspective on the scope and development of the field.

The literature in this area varies considerably in the technologies used, target populations, study designs, application purposes, and outcomes assessed. Mapping the evidence according to the functions performed by AI and the stage of evidence development helps distinguish forms of research that would otherwise fall within the same broad field. Technical development and validation, feasibility and pilot testing, and studies evaluating intervention outcomes do not represent equivalent stages of evidence. A function-based approach allows the scope of current applications to be considered alongside the maturity of research supporting them. The aim of this scoping review was to map the available evidence on AI-supported exercise and physical activity applications from a public health perspective, classify the main functions of AI in this field, describe research trends, and identify methodological gaps in the existing literature.

## Materials and methods

### Research design

This study was designed as a scoping review to systematically map the literature on AI-supported exercise and physical activity applications, identify key characteristics and research trends, examine gaps in the evidence, and organize methodologically heterogeneous studies within a common analytical framework. The review was conducted in accordance with the Preferred Reporting Items for Systematic Reviews and Meta-Analyses Extension for Scoping Reviews (PRISMA-ScR) reporting guideline (19) and the Joanna Briggs Institute (JBI) methodology for scoping reviews (20). The review was not prospectively registered.

### Procedures

The scoping review process comprised the following stages: formulation of the research questions, definition of the eligibility criteria, identification of information sources and the search strategy, literature searching, selection of eligible studies, data extraction and charting, classification of the functional roles of AI applications and stages of evidence development, synthesis of the findings, and reporting of the results.

### Research questions

The review was guided by five research questions developed to map the scope of the available evidence, characterize the functional roles of AI applications, and identify key research gaps in the literature (Table 1).

**Table 1:** Research questions.

Research question
1. How are studies investigating AI-supported exercise and physical activity applications distributed by publication year and country?
2. What are the primary functional roles of AI in current exercise and physical activity applications (e.g., Intervention Delivery or Adaptation, Monitoring or Assessment, Prediction or Risk Stratification, Professional Decision Support, and System and Environmental Planning)?
3. In which target populations and clinical or community-based settings have these AI applications been investigated?
4. What study designs, intervention characteristics, and outcome types have been used to evaluate these applications?
5. What is the current stage of evidence development for these applications (e.g., technical development/validation, feasibility or acceptability, pilot or short-term intervention evaluation), and what key methodological and evidence gaps remain in relation to their broader translation into clinical and public health practice?

### Data sources

The literature search was conducted in Web of Science, Scopus, and PubMed to identify eligible studies on AI-supported exercise and physical activity applications. The search was limited to studies published between 2015 and 2025. The advanced search functions of each database were used to construct the search using the Boolean operators AND and OR. The core search string was (“artificial intelligence”) AND (“physical activity” OR “exercise”) AND (“public health” OR “health promotion”). Queries were adapted to the relevant title, abstract, keyword, or topic fields according to the search functions available in each database. Detailed database-specific search strategies are provided in the supplementary material. The initial search identified 612 records, 172 from Web of Science, 221 from Scopus, and 219 from PubMed.

### Eligibility criteria

The inclusion and exclusion criteria were defined a priori to ensure a transparent and consistent study selection process. The criteria are presented in Table 2.

**Table 2:** Inclusion and exclusion criteria.

Inclusion	Exclusion
• Studies published in peer-reviewed scientific journals	• Conference proceedings, theses, books, or book chapters
• Full-text articles	• Articles without accessible full text
• Articles published in English	• Articles published in languages other than English
• Completed empirical studies in which AI was directly applied to exercise, physical activity, or related health applications	• Studies in which AI was not directly applied to exercise, physical activity, or related health applications
• Studies reporting original empirical findings relevant to the research questions of the review	• Review articles, editorials, commentaries, viewpoint/perspective papers, or retracted articles
• Studies published between 2015 and 2025	• Duplicate records
	• Study protocols

### Study selection

Records retrieved from the three databases were combined, and duplicate records were removed. The titles and abstracts of the remaining records were independently screened by two reviewers against the predefined eligibility criteria. Full texts of potentially eligible studies were then assessed, and studies meeting the inclusion criteria were included in the final synthesis. Both title and abstract screening and full-text eligibility assessment were conducted independently by two reviewers, with disagreements regarding study eligibility resolved through discussion and consensus. A total of 24 studies were included in the scoping review. Reporting followed the PRISMA-ScR guideline, and the study selection process is presented in Figure 1 using the PRISMA 2020 flow diagram.

**Figure 1:**
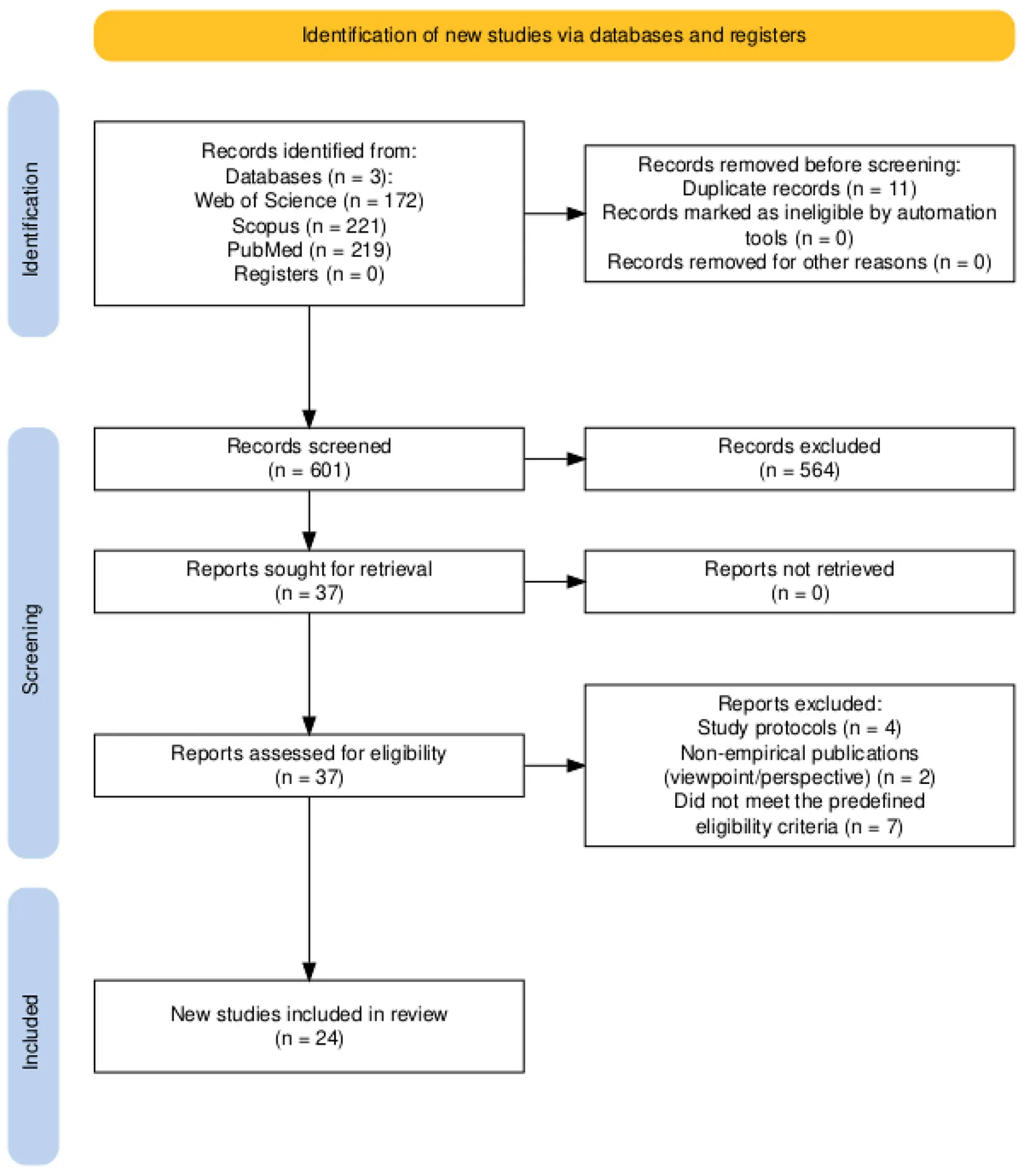
PRISMA 2020 flow diagram of the study selection process. This figure was generated using the PRISMA2020 Shiny App with data from the present review.

### Data extraction and charting

Data were systematically extracted and charted using a predeveloped standardized data extraction form. For each study, the following information was recorded: author(s), publication year, journal, country/region, target population, study setting, study design, sample size, AI technology or method, primary and, where applicable, secondary functional role of AI, research focus, key findings, and stage of evidence development. The extracted data were organized to facilitate comparison of methodological and application-related differences across studies and enable the function-based classification of AI applications.

### Data synthesis and classification

The included studies were heterogeneous in terms of study design, target population, AI methods, and outcomes assessed. The findings were therefore synthesized using a descriptive, function-based approach. AI applications were classified according to the primary purpose of AI and the role it performed within each study. Five functional categories were identified: *Intervention Delivery or Adaptation, Monitoring or Assessment, Prediction or Risk Stratification, Professional Decision Support*, and *System and Environmental Planning*. When an application served more than one function, the role most directly related to the primary research objective was classified as the primary function, and other relevant roles were recorded as secondary functions.

Studies were also classified according to their stage of evidence development, based on the research objective, study design, and type of evaluation conducted. The stages were used to characterize the levels of research represented in the literature, ranging from technical or conceptual development and validation to prototype and user evaluation, feasibility and proof-of-concept studies, pilot or comparative intervention evaluations, and prediction model development or validation. This classification was not intended as a hierarchical assessment of methodological quality, but as an analytical tool for describing where each study was positioned within the development and evaluation of the evidence. Classification of functional roles and stages of evidence development was conducted jointly by two reviewers, with differences in classification discussed and resolved by consensus.

## Results

The key characteristics of the 24 studies included in the final synthesis, including the functional roles of AI, stages of evidence development, outcome types assessed, and key findings, are presented in Table 3.

**Table 3:** Functional mapping of included studies by AI role, evidence-development stage, and outcomes.

Study	Population/ setting	Study design	AI technology	Role of AI	Stage of evidence development	Outcome type	Key findings
Anan et al. (21)	Japan; employees aged 20–64 years with frequent neck/shoulder pain or stiffness and/or low back pain in a company setting. 121 were randomized; 94 were analyzed (48 intervention, 46 control).	Two-arm, unblinded randomized controlled trial; 12-week intervention.	AI-assisted interactive health-promotion chatbot (Secaide v0.9) delivered through LINE; tailored replies based on user responses.	Primary: Intervention Delivery or Adaptation	Randomized clinical effectiveness evaluation	Clinical outcomes; adherence	The AI-assisted exercise program was associated with greater improvement in neck/shoulder and low-back symptoms than usual care, with 92% intervention adherence. The trial did not isolate the added effect of AI from the exercise/reminder package.
Areias et al. (36)	United States; 6,125 patients enrolled in a remote digital musculoskeletal care program for low back pain.	Prognostic model-development study using prospectively collected digital-care data.	Recurrent neural networks (RNNs), LightGBM, and SHAP explainability using dynamically updated clinical, exercise-performance, and contextual data.	Primary: Prediction or Risk Stratification; Secondary: Professional Decision Support	Predictive model development and validation	Predictive performance; clinical decision support	AI predicted the likelihood of clinically significant pain response throughout the intervention, with moderate discrimination by session 7 (ROC-AUC = 0.70 for RNN and 0.71 for LightGBM). Predictions were designed to flag patients at risk of nonresponse and support physical therapists in considering treatment re-tailoring.
Chew et al. (32)	Singapore; adults participating in the eTRIP weight-management program. 251 enrolled and 230 completed the 7-day evaluation period.	Single-group pretest–posttest mixed-methods evaluation	AI-assisted eTRIP app: chatbot-based check-ins, food-based computer vision image recognition, and automated time-based nudges	Primary: Monitoring or Assessment; Secondary: Intervention Delivery or Adaptation	Feasibility and preliminary effectiveness evaluation	Behavioral outcomes; user experience/ feasibility	After the 1-week program, significant improvements were observed in overeating and snacking habits, self-regulation of eating behavior, depressive symptoms, and physical activity. Total physical activity increased by 1,288.60 MET-min/week (*P* < 0.001). The single-group design and short follow-up preclude attribution of these changes specifically to the AI components or conclusions regarding their sustainability.
Davis et al. (22)	Australia; 28 community-dwelling adults aged 45–75 years participating in a pilot physical-activity and diet program.	Secondary process evaluation of a 12-week single-arm repeated-measures pilot study	IBM Watson-based natural language processing virtual health assistant (“Paola”)	Primary: Intervention Delivery or Adaptation	Process, acceptability, and engagement evaluation	Technical performance; engagement; adherence; acceptability	Paola correctly asked scripted questions and provided appropriate responses during weekly check-ins 97% and 96% of the time, respectively, but correctly responded to only 21% of spontaneous exchanges. Participants completed 63% of weekly check-ins and met their step goals approximately 59% of the time. Engagement with Paola was not significantly associated with changes in MVPA.
Deng et al. (42)	China; college-student physical-health context. The study used a self-developed questionnaire, expert surveys, and literature-based system development; the student sample size is not clearly reported in the article.	Questionnaire-based cross-sectional study combined with development of a conceptual intelligent governance system	Big data analytics, machine learning, artificial neural networks, and AI-supported data processing within an intelligent governance system	Primary: System and Environmental Planning; Secondary: Monitoring or Assessment	Applied system/ framework development	System/governance outcomes	The study identified individual, family, school, and societal factors associated with declining physical health among college students and proposed a three-level intelligent governance system integrating infrastructure, data resources and processing technologies, and intelligent service applications. The proposed system incorporates physical health monitoring, venue optimization, personalized exercise guidance, and decision support, although these AI-enabled functions remained largely conceptual and were not empirically evaluated.
Dergaa et al. (6)	Five hypothetical patient profiles with diverse health conditions and fitness goals	Simulation study with expert evaluation	OpenAI GPT-4 large language model	Primary: Intervention Delivery or Adaptation; Secondary: Professional Decision Support	Simulation-based expert evaluation	Prescription quality; safety; individualization	GPT-4 generated generally safety-conscious 30-day exercise programs for five hypothetical profiles; however, the prescriptions lacked sufficient precision in addressing individual health conditions and fitness goals and tended to prioritize safety over training effectiveness. The authors concluded that GPT-4 may serve as a supplementary tool for exercise prescription but should not substitute for individualized, progressive, and condition-specific guidance from healthcare and fitness professionals.
Figueroa et al. (23)	United States; 18 low-income women aged 27–41 years (10 Spanish-speaking, 8 English-speaking), recruited through SNAP-Ed in California and Tennessee.	Qualitative user-centered design pilot study with prototype testing, Wizard of Oz testing, and a co-design workshop	IBM Watson Assistant-based text-message conversational agent (chatbot)	Primary: Intervention Delivery or Adaptation	Prototype development/ user testing	User experience; acceptability; design/ equity considerations	After testing the prototype, most participants reported positive perceptions of physical activity chatbots and valued their potential for support and motivation. Important design considerations included personalization, digital literacy, linguistic and cultural appropriateness, privacy, flexibility, and family inclusion. The study evaluated user perceptions and design requirements; the effectiveness of the chatbot in increasing physical activity was not assessed.
Forte et al. (37)	Portugal; 654 adolescents aged 10–19 years (334 male, 320 female) from the FITescola project.	Observational cross-sectional study with neural-network model development and internal validation	Deep-learning neural network (NNET)	Primary: Prediction or Risk Stratification	Model development and internal validation	Predictive performance	The neural network classified obesity risk based on BMI percentiles and physical fitness variables with 75% accuracy and an ROC-AUC of 0.64. 10-fold cross-validation yielded 71% accuracy and an ROC-AUC of 0.66. Lower physical fitness was associated with greater obesity risk, but the model was validated only internally and was not evaluated in independent populations.
Guo (33)	Human-pose image datasets (MPII Human Pose, COCO Keypoints, 3DPW, Human3.6M) with additional real-case testing	Technical model development and validation study using public human-pose datasets and real-case testing	Computer vision-based multi-scale 3D human-body reconstruction using OpenPose, Pix2Pix, channel attention, pixel-aligned implicit functions, and neural-network components	Primary: Monitoring or Assessment	Technical development and validation	Technical performance	The proposed multi-scale 3D human-body reconstruction model showed improved reconstruction performance compared with the evaluated comparison models and achieved real-case reconstruction accuracy ranging from 92.22% to 94.98% across six tests. The study showed that the approach was technically feasible for online physical-education assessment but did not evaluate effects on physical activity, learning outcomes, or health outcomes.
Hassoon et al. (24)	United States; 42 physically inactive, overweight or obese cancer survivors randomized equally to MyCoach, SmartText, or control.	Three-arm randomized controlled pilot trial	AI-based recommendation systems: voice-assisted MyCoach delivered via Amazon Echo/Alexa and autonomous SmartText coaching delivered via text messaging, integrated with wearable-sensor data	Primary: Intervention Delivery or Adaptation; Secondary: Monitoring or Assessment	Pilot randomized efficacy evaluation	Behavioral outcome (objective physical activity); safety	MyCoach increased daily steps by 3,618.2 steps/day over the 4-week intervention. The increase was significantly greater than control (net difference = 3,568.9 steps/day, 95% CI 1,483–5,655; *p* < 0.001) and SmartText (net difference = 2,160.6 steps/day, 95% CI 11–4,310; *p* = 0.049). SmartText did not significantly differ from control. The trial was small and short-term, limiting conclusions about longer-term effectiveness and generalizability.
Hautala et al. (38)	Finland; 71 patients referred after acute coronary syndrome and participating in 12-month exercise-based cardiac rehabilitation.	Secondary predictive modeling study using data from an exercise-based cardiac rehabilitation trial	Machine learning–based feature importance and predictive modeling (cross-decomposition methods and linear regression)	Primary: Prediction or Risk Stratification; Secondary: Professional Decision Support	Model development/ internal evaluation	Predictive performance; health-economic outcomes	Machine-learning-based risk-factor ranking identified diabetes as the strongest predictor of 12-month health care costs among patients participating in exercise-based cardiac rehabilitation, explaining 16.4% of cost variance. Adding BMI and systolic blood pressure increased the model's explained variance to 18.2%, while the full 13-factor model explained 30.7%. The findings suggest potential utility for risk assessment and health care resource planning, but the small sample and lack of external validation limit generalizability.
Itoh et al. (25)	Japan; 99 patients with chronic low back pain receiving pharmacological treatment (48 exercise-app group, 51 conventional-care group).	Multicenter, open-label, randomized parallel-group trial	AI-assisted chatbot delivered through the Secaide mobile messaging app (LINE)	Primary: Intervention Delivery or Adaptation	Comparative effectiveness/ randomized trial	Clinical outcomes; work productivity; quality of life	The AI-assisted mobile exercise and education intervention did not significantly improve the primary outcome of work productivity compared with conventional care (*p* =.43). However, significant between-group improvements were observed in subjective low-back-pain symptoms, health-related quality of life (EQ-5D-5L), and kinesiophobia at 12 weeks. The intervention combined AI-assisted messaging, patient education, exercise therapy, and usual pharmacological care; therefore, the observed effects cannot be attributed specifically to the AI component.
Lai et al. (34)	Taiwan; laboratory-scale lower-limb rehabilitation exercise data from two test subjects.	Technical system development and validation study	AIoT-based wearable-sensor system using SVM and ANFIS machine-learning models with HHT-based feature extraction	Primary: Monitoring or Assessment; Secondary: Professional Decision Support	Technical development and internal validation	Technical performance; rehabilitation monitoring	The AIoT-based rehabilitation recognition system demonstrated high technical performance in recognizing six lower-limb rehabilitation exercises. Several SVM and ANFIS feature patterns achieved up to 0.99 accuracy in 5-fold cross-validation, and selected models were further tested using data from a second subject. The findings indicate the technical feasibility of wearable-sensor-based rehabilitation monitoring.
Li and Li (26)	China; 100 residents aged 20–45 years from two units (A and B) assessed for physical fitness and chronic-disease characteristics.	System development and empirical evaluation study	Back-propagation neural network (BPNN)-based AI decision-support system with rule-based reasoning	Primary: Intervention Delivery or Adaptation	System development/ preliminary empirical evaluation	Physical-fitness assessment; exercise-prescription decision support	The BPNN-based AI decision-support system integrated physical fitness and health data to generate individualized physical-fitness evaluations and exercise guidance. The system was designed to select exercise prescriptions according to individual health status and incorporated chronic-disease information into decision support. The study primarily reports system development and preliminary application; comparative clinical effectiveness was not evaluated.
Maher et al. (27)	Australia; 31 inactive community-dwelling adults aged 45–75 years.	12-week single-arm pre–post proof-of-concept study	IBM Watson–based natural language processing virtual health coach (“Paola”) integrated with a wearable activity tracker	Primary: Intervention Delivery or Adaptation	Proof-of-concept/ preliminary effectiveness evaluation	Behavioral outcomes; feasibility; anthropometric outcomes	Over 12 weeks, weekly physical activity increased by 109.8 min (95% CI 1.9–217.7; *p* = 0.005), Mediterranean diet adherence improved by 5.7 points (95% CI 4.2–7.3; *p* < 0.001), body weight decreased by 1.3 kg, and waist circumference decreased by 2.1 cm; blood pressure did not significantly change. Recruitment and retention were high, with 90% retention at 12 weeks and no adverse events. The single-arm design prevents the observed changes from being attributed specifically to the AI component.
Marcuzzi et al. (28)	Norway; 294 adults with neck and/or low back pain referred to specialist multidisciplinary outpatient care.	Three-arm randomized clinical trial with 6-month follow-up	Knowledge-based AI decision-support system using case-based reasoning (SELFBACK smartphone app)	Primary: Intervention Delivery or Adaptation	Comparative effectiveness/ randomized clinical evaluation	Clinical outcomes	The individually tailored AI-based SELFBACK app, when added to usual care, did not significantly improve the primary outcome of musculoskeletal health at 3 months compared with either usual care alone or non-tailored web-based self-management support. Most secondary outcomes also showed no significant between-group differences at 3 months; Global Perceived Effect was the exception in the comparison with usual care. App uptake was suboptimal, with 29.3% of participants assigned to the app group never accessing the intervention.
Meng et al. (39)	China; community-dwelling frail adults aged ≥65 years. 243 were enrolled and 150 completed the 24-week three-group exercise experiment.	Double-blind, three-arm randomized controlled trial with machine-learning-based predictive modeling	Machine-learning classification models with stacking ensemble and SHAP-based explainability	Primary: Prediction or Risk Stratification	Predictive model development/ internal validation	Clinical outcomes; predictive performance	Following the 24-week exercise intervention, 69 of 150 participants (46.0%) transitioned from frail to non-frail status. The stacking model predicted post-intervention frailty status with 67.8% accuracy and an F1-score of 71.3% (AUC = 0.711). SHAP analysis identified 10-m maximum walking speed as the most influential physical-fitness feature for prediction. The model was internally evaluated and was not externally validated.
Omarov et al. (35)	Kazakhstan; video data of physical-culture students covering five exercise categories (pull-ups, push-ups, squat, biceps, and neck workout), with 100 min of video per class.	Technical model development and validation study	PoseNet-based pose estimation with convolutional/ deep neural network for skeleton-based exercise classification	Primary: Monitoring or Assessment	Technical development and internal validation	Technical performance	The PoseNet-enabled deep neural network classified and monitored exercise movements in real time, achieving approximately 98% accuracy after 100 training epochs. The findings indicate technical feasibility for real-time exercise monitoring and feedback, but effects on physical activity behavior, exercise adherence, performance, or health outcomes were not evaluated.
Persell et al. (29)	United States; 333 adults with uncontrolled hypertension were randomized; 297 completed 6-month follow-up.	Two-group, open-label randomized clinical trial with 6-month follow-up	Conversational AI smartphone coaching application using proprietary algorithms and tailored behavioral coaching	Primary: Intervention Delivery or Adaptation	Comparative effectiveness/ randomized clinical evaluation	Clinical outcomes; self-management/ behavioral outcomes	The AI-based coaching app did not significantly reduce systolic blood pressure at 6 months compared with the tracking app plus home blood pressure monitoring (adjusted difference −2.0 mmHg, 95% CI −4.9 to 0.8; *p* = 0.16). Self-reported physical activity was 26.7 min/week higher in the intervention group, but the between-group difference was not statistically significant (95% CI −5.4 to 58.8; *p* = 0.10). The intervention significantly improved self-confidence in controlling blood pressure, while no significant differences were observed for the other secondary outcomes.
Philuek et al. (30)	Thailand; 9 healthy overweight male university students aged ≥18 years (6 ChatGPT-program, 3 control).	Pilot randomized controlled intervention study	ChatGPT-4.0–generated exercise program	Primary: Intervention Delivery or Adaptation	Pilot comparative intervention evaluation	Anthropometric and physical-fitness outcomes	After the 8-week intervention, the ChatGPT-generated exercise-program group had significantly lower BMI and post-exercise heart rate and higher 30-second sit-to-stand performance than the control group. No significant between-group differences were observed for several other outcomes, including body weight, body fat, skeletal muscle measures, handgrip strength, flexibility, and lung capacity. Given the very small sample (n=9), the findings represent preliminary evidence and require confirmation in larger studies.
Shao et al. (43)	Shanghai, China; 609,000 street-view images within the study area and 128 valid online questionnaire responses from residents (mean age 29 years).	Cross-sectional mixed-methods environmental assessment using street-view image analysis, GIS-based clustering, and an online questionnaire	DeepLab V3+-based semantic segmentation of street-view images, combined with GIS and cluster analysis	Primary: System and Environmental Planning	Environmental assessment/ exploratory planning application	Environmental/ spatial outcomes; perceived health opportunity; equity	AI-based streetscape analysis revealed spatial inequalities in health-promotive urban environments across Shanghai. Streets with lower health ratings were more often characterized by lower vegetation visibility and higher building density, while greener streetscapes were generally rated as more supportive of physical, mental, and social health. The study identified environmental features relevant to walking, jogging, cycling, and other physical activities, but did not directly measure actual physical activity behavior or health outcomes.
Wei et al. (40)	China; 90 adults aged 60–75 years with sarcopenia, allocated to Yi Jin Jing plus resistance training, resistance training, or control (30 per group).	Randomized controlled trial with post-intervention machine-learning prediction modeling	Explainable machine learning; stacking ensemble with SHAP-based feature interpretation	Primary: Prediction or Risk Stratification	Prediction model development and internal validation embedded within an RCT	Clinical outcomes; predictive performance	Following the 24-week intervention, the stacking model predicted post-intervention sarcopenia status with the highest accuracy (85.7 ± 10.6%) and F1 score (75.3 ± 11.5%) among the evaluated models. The exercise intervention itself improved several muscle-related outcomes, but these intervention effects should be distinguished from the predictive performance of the AI model.
Wu et al. (31)	China; 61 adults aged 24–58 years (32 men, 29 women) undergoing incremental cardiopulmonary exercise testing on cycling equipment.	Experimental machine-learning model development and validation study	Machine-learning regression with iterative feature selection; final ridge regression model	Primary: Intervention Delivery or Adaptation	Model development and internal validation	Technical prescription performance	The machine-learning approach predicted the individual heart rate–wattage relationship from static body data and enabled calculation of cycling-equipment wattage required to achieve a personalized target heart rate. Ridge regression provided the best predictive performance; the final model achieved an adjusted R^2^ of 0.7068 in the test set, increasing to 0.8247 after exclusion of two high-error cases. The model showed technical feasibility for AI-supported personalized exercise-intensity prescription, but the study did not evaluate whether its use improved physical activity behavior, adherence, fitness, or health outcomes.
Xu et al. (41)	China; 10 real patient cases/patient profiles whose prescriptions were subsequently evaluated by 24 experts	Mixed-methods comparative evaluation of AI-generated exercise prescriptions	ChatGPT-4.0 and Intelligent Health Promotion System (IHPS)	Primary: Professional Decision Support	Comparative expert evaluation of decision-support systems	Prescription accuracy, comprehensiveness, applicability, and safety	ChatGPT-4.0 generally received higher expert ratings than IHPS for the accuracy, comprehensiveness, and applicability of personalized exercise prescriptions. However, safety concerns emerged for patients with functional movement limitations, and both systems showed limitations in medication-related guidance and dynamic adaptation. The findings indicate a potential supplementary role for AI in professional exercise-prescription decisions but do not establish its suitability for autonomous clinical use.

AI, artificial intelligence; AIoT, artificial intelligence of things; ANFIS, adaptive neuro-fuzzy inference system; BPNN, back-propagation neural network; CBT, cognitive behavioral therapy; CNN, convolutional neural network; ECR, exercise-based cardiac rehabilitation; GIS, geographic information system; IHPS, Intelligent Health Promotion System; IoT, Internet of Things; LightGBM, light gradient boosting machine; ML, machine learning; MLP, multilayer perceptron; RNN, recurrent neural network; RSMI, relative skeletal muscle mass index; SHAP, SHapley Additive exPlanations; SVM, support vector machine.

### Publication year and country distribution

The studies included in this review were published between 2020 and 2025. Four studies were published in 2020, four in 2021, five in 2022, three in 2023, seven in 2024, and one in 2025. Publication activity was highest in 2024 (Figure 2). Across the period examined, the number of publications varied from year to year rather than showing a consistent upward trend.

**Figure 2:**
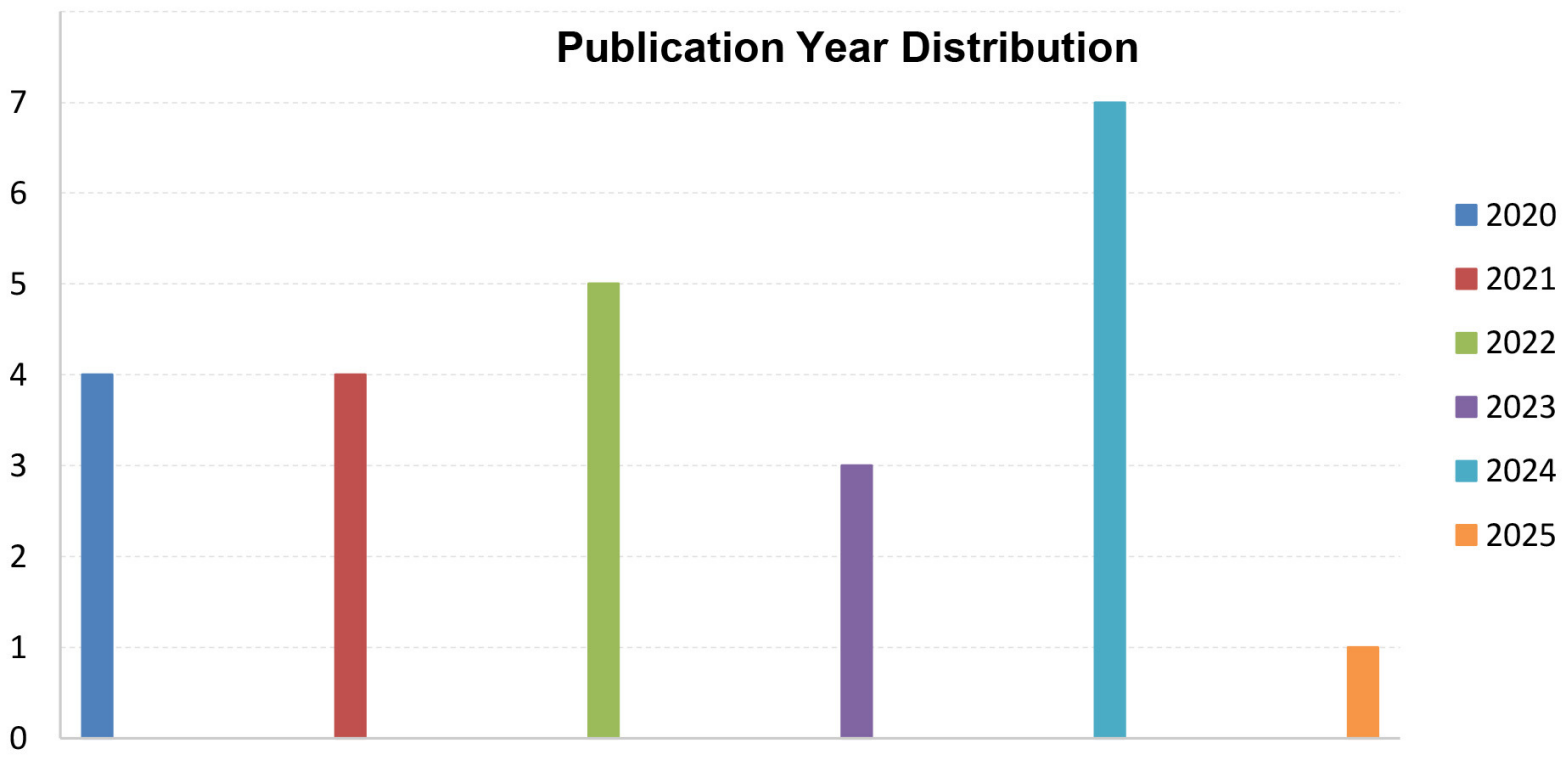
Distribution of the included studies by publication year.

China accounted for the largest number of included studies (*n* = 8), followed by the United States (*n* = 4). Australia and Japan were represented by two studies each, while Qatar, Singapore, Norway, Taiwan, Kazakhstan, Thailand, Finland, and Portugal were represented by one study each (Figure 3).

**Figure 3:**
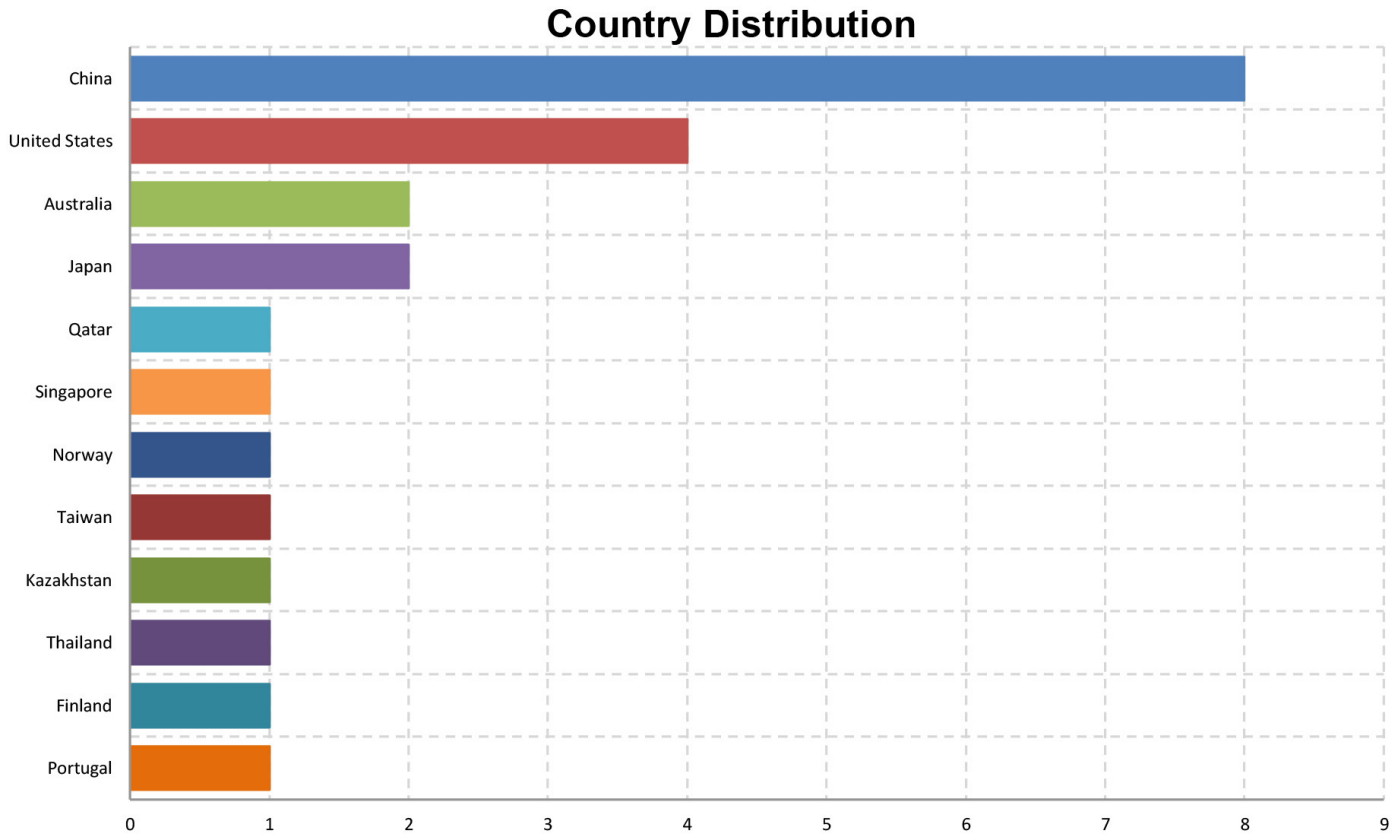
Geographical distribution of the included studies.

### Study characteristics and evidence development profile

The included studies covered a broad range of target populations and application settings. Clinical populations included individuals with chronic low back or neck pain, adults with uncontrolled hypertension, cancer survivors, older adults with frailty or sarcopenia, and patients participating in cardiac rehabilitation. Other studies involved healthy or community-dwelling adults, adolescents, university students, low-income women, and participants in physical education settings. Some studies used hypothetical patient profiles, human movement datasets, or urban environmental data and did not involve participant-based interventions. Accordingly, the studies represented a range of contexts, including clinical and rehabilitation settings, community- and home-based digital applications, educational settings, laboratory environments, and environmental assessments.

Research designs and outcomes assessed also varied considerably across the included studies. The included studies comprised randomized controlled interventions, single-group pretest–posttest and proof-of-concept studies, mixed-methods and user-centered evaluations, cross-sectional studies, technical system and model development or validation studies, prediction model development studies, simulations and expert evaluations, and environmental assessment studies. Outcomes included clinical and physical fitness measures, physical activity behavior, technical classification or model performance, predictive accuracy, user experience and acceptability, intervention adherence, the quality and safety of exercise prescriptions, health care costs, and environmental or spatial indicators. This range of designs and outcomes reflects different stages in the development and evaluation of AI applications and therefore does not constitute a single body of evidence addressing intervention effectiveness.

When the stages of evidence development were examined, the studies ranged from technical or conceptual system development and internal validation to prototype and user testing, feasibility and proof-of-concept evaluations, and pilot or comparative randomized interventions. Most prediction-focused studies remained at the model-development and internal-validation stage. Some intervention studies, by contrast, directly evaluated clinical or behavioral outcomes using comparative designs. Independent external validation and long-term real-world evaluation were also absent from several technical and predictive models. The available literature thus represents AI-supported exercise and physical activity applications at different stages of evidence development.

### Function-based synthesis of artificial intelligence applications

The included studies were synthesized according to the primary functions performed by AI in the public health context. Classification was based on the role performed by AI within exercise and physical activity applications, independent of study design or target population. Five functional categories were identified: (1) Intervention Delivery or Adaptation, (2) Monitoring or Assessment, (3) Prediction or Risk Stratification, (4) Professional Decision Support, and (5) System and Environmental Planning. Each study was assigned to a category according to the primary function of AI, with secondary functions recorded when an application served more than one role. This classification provided a consistent basis for mapping studies with different designs and application contexts according to the primary purpose for which AI was used.

### Intervention delivery or adaptation

This category included studies in which AI was directly used to develop, personalize, adapt, or deliver exercise and physical activity interventions. Applications included generating personalized exercise prescriptions, determining exercise intensity based on individual characteristics, providing digital or virtual coaching, and supporting physical activity behavior (6, 21–31).

Intervention outcomes varied across studies. Hassoon et al. (24) reported a significant increase in daily step count in the AI-supported voice coaching group compared with the control group. Persell et al. (29) found a higher weekly duration of physical activity in the AI-based coaching group, although the between-group difference was not statistically significant. The individually tailored AI-based SELFBACK intervention evaluated by Marcuzzi et al. (28) showed no significant advantage over usual care or non-tailored web-based support for the primary musculoskeletal outcome. Anan et al. (21) reported differences favoring the intervention group for some clinical outcomes. In Itoh et al. (25), no significant between-group difference was found for the primary outcome of work productivity; several secondary outcomes, including low back pain symptoms, health-related quality of life, and kinesiophobia, favored the intervention group. Maher et al. (27) reported an increase in physical activity following a single-group intervention.

Other studies focused on AI-supported personalization, exercise program generation, or application development without directly evaluating intervention outcomes. Wu et al. (31) developed a machine-learning model to predict the exercise intensity corresponding to a target heart rate based on individual characteristics. Dergaa et al. (6) evaluated exercise programs generated by GPT-4 for hypothetical patient profiles, while Philuek et al. (30) examined an eight-week exercise program generated in advance by ChatGPT-4.0 in a small pilot randomized study. Figueroa et al. (23) focused on the user-centered development of an AI-supported physical activity coach, whereas Davis et al. (22) evaluated the performance of a virtual health assistant during structured and spontaneous user interactions.

### Monitoring or assessment

This category included studies in which AI was used to monitor physical activity and exercise, assess movement or exercise performance, and process behavioral data. Computer vision, wearable sensors, machine-learning methods, and AI-supported digital systems were used for movement classification, exercise performance assessment, modeling of human body movement, and monitoring of physical activity-related behaviors (32–35).

Some studies focused primarily on the technical performance of the systems. Omarov et al. (35) reported approximately 98% accuracy for a model developed for real-time exercise classification. Lai et al. (34) compared different machine-learning models using sensor data and achieved classification accuracy of up to 0.99 with selected models. Guo (33) evaluated the technical performance of an AI-based approach to three-dimensional human body reconstruction and movement assessment in an exercise context. In a short-term single-group evaluation, Chew et al. (32) reported changes in several behavioral outcomes, including physical activity, following use of an AI-supported application.

### Prediction or risk stratification

This category included studies in which AI was used to predict clinical outcomes, health risks, or post-intervention status related to physical activity and exercise. Machine-learning models were used to classify obesity risk, predict clinical response to digital musculoskeletal programs, identify risk factors associated with health care costs in cardiac rehabilitation, and predict frailty or sarcopenia status following exercise interventions (36–40).

Areias et al. (36) developed a model to predict the likelihood of clinical response among individuals participating in a digital musculoskeletal program, while Forte et al. (37) focused on classifying obesity risk using physical fitness indicators. Hautala et al. (38) used machine learning to identify factors associated with health care costs in the context of cardiac rehabilitation. Meng et al. (39) and Wei et al. (40) developed models using data from randomized exercise trials to predict post-intervention frailty and sarcopenia status, respectively. In both studies, AI was used to predict post-intervention outcomes and was not involved in delivering the exercise intervention.

### Professional decision support

This category included studies in which AI was used to support health professionals in exercise prescription and clinical assessment. Xu et al. (41), whose primary AI function was classified as Professional Decision Support, compared personalized exercise prescriptions generated by ChatGPT-4.0 and the Intelligent Health Promotion System (IHPS) for hypothetical cases involving hypertension with different coexisting health conditions. The prescriptions generated by ChatGPT-4.0 generally received higher expert ratings for accuracy, comprehensiveness, and applicability, although safety concerns were identified in some cases involving functional movement limitations. Limitations were also reported for both systems in medication-related guidance and dynamic adaptation of exercise programs. The AI-generated prescriptions were not implemented in actual patients or evaluated for clinical outcomes. The findings reflect expert evaluation of recommendations generated by AI-supported systems for professional exercise prescription; clinical effectiveness was not evaluated.

### System and environmental planning

This category included studies in which AI was used beyond individual-level exercise and physical activity interventions to support system development and the assessment of environmental characteristics related to physical activity. The two studies in this category applied AI to system- and environment-level applications at different scales (42, 43).

Deng et al. (42) developed an intelligent governance system for the physical health management of university students that incorporated individual, family, school, and societal components. Within the proposed system, AI was associated with functions such as physical health monitoring, facility and resource optimization, personalized exercise guidance, and decision support. Many of these AI functions, however, were addressed primarily at the level of system development and conceptual modeling.

Shao et al. (43) used deep learning-based image analysis to assess features of the urban environment related to physical activity. Large-scale street-view images were analyzed to examine the spatial distribution of environmental characteristics. Environments with lower health ratings were reported to have characteristics such as lower visible greenery and higher building density. Actual physical activity behavior and health outcomes were not directly measured.

## Discussion

This scoping review examined AI-supported exercise and physical activity applications through a function-based public health framework. The 24 studies covered five functional areas: intervention delivery or adaptation, monitoring or assessment, prediction or risk stratification, professional decision support, and system and environmental planning. The maturity of the research varied across these areas, ranging from technical development and validation to pilot applications, predictive modeling, and randomized controlled interventions. Potential applications of AI should be distinguished from demonstrated improvements in clinical or public health outcomes.

The range of functions identified here is consistent with earlier research showing that AI can serve different purposes in physical activity research. An et al. (12), for example, identified applications involving the recognition of behavioral patterns, prediction of outcomes, and development of personalized physical activity interventions. The distinction lies in how the evidence is organized: the present review examines applications according to their functional roles in public health and stages of evidence development, while previous classifications have focused more directly on the AI methodologies employed.

The socioecological approach provides a useful framework for interpreting these functional roles within public health. McLeroy et al. (44) conceptualized health behavior as being shaped by influences at individual, interpersonal, organizational, community, and policy levels. Golden and Earp (45) described social ecological models as a means of understanding interactions between individuals and the broader environments in which health behavior occurs. The functional categories identified here were not derived from the Socioecological Model; the model provides a framework for locating the identified applications within the broader public health context. Intervention delivery or adaptation primarily addresses individual-level personalization, whereas monitoring, risk prediction, and professional decision support connect individual data with health-service processes. System and environmental planning extends to environmental and broader system-level applications. Evidence remains concentrated at the individual and clinical levels, with comparatively few studies addressing environmental or system-level uses of AI. Social ecological research has documented a comparable imbalance, with health promotion interventions historically concentrated at individual and interpersonal levels (45).

For studies classified under Intervention Delivery or Adaptation, AI was used to generate exercise prescriptions, tailor interventions to individual characteristics, and provide behavioral support through digital coaching (6, 21–31). Across these studies, the capacity to personalize an intervention did not necessarily translate into greater effectiveness. Hassoon et al. (24), for example, reported a significant increase in daily step count relative to the control group in a short-term pilot randomized trial of voice-based AI coaching. Persell et al. (29) found 26.7 more minutes of physical activity per week in the AI coaching group, but the between-group difference was not statistically significant. Marcuzzi et al. (28) likewise found no significant advantage of the individually tailored AI-based SELFBACK intervention over usual care or non-tailored web-based support for the primary musculoskeletal outcome.

The same distinction between personalization and effectiveness is apparent in the remaining intervention studies. Anan et al. (21) reported favorable differences in some clinical outcomes. In Itoh et al. (25), no significant benefit was observed for the primary outcome of work productivity, although the intervention group showed improvements in several secondary outcomes, including low back pain symptoms, health-related quality of life, and kinesiophobia. Both interventions combined AI-supported messaging with other components such as exercise, education, reminders, or usual care, making it difficult to isolate the contribution of AI itself. Maher et al. (27) reported an increase in physical activity in a single-arm study, but the absence of a comparison group limits causal interpretation. The intervention literature thus indicates possible benefits under some conditions without establishing a consistent added benefit of AI-based personalization over simpler digital support or usual care. This interpretation is broadly consistent with a recent systematic review describing AI-based chatbots as potentially personalized and scalable tools for promoting physical activity, although intervention evidence in this area remains limited (46).

Large language models and generative AI make the distinction between personalization and demonstrated effectiveness particularly clear. Dergaa et al. (6) found that GPT-4 generally produced exercise programs that prioritized safety for hypothetical patient profiles, although the prescriptions were less precise when individual health conditions and fitness goals were considered. In the nine-participant pilot randomized study by Philuek et al. (30), an eight-week program generated in advance by ChatGPT-4.0 differed from the control condition on some anthropometric and physical fitness outcomes; several other outcomes showed no between-group differences. ChatGPT did not monitor participants or adjust the program during the intervention. A previous systematic review also identified potential for personalization and scalability in generative AI-based exercise and training prescription but reported limitations in applicability, reliability, and adaptability (47). Current research positions generative AI as an early-stage tool for exercise prescription, with professional evaluation becoming increasingly important as clinical complexity increases (6, 30, 41).

A different evidence profile emerges for Monitoring or Assessment, where AI has primarily been evaluated for movement recognition, exercise performance assessment, rehabilitation monitoring, and automated processing of behavioral data (32–35). Omarov et al. (35) reported approximately 98% accuracy in real-time exercise classification, while Lai et al. (34) achieved classification accuracy of up to 0.99 with selected sensor and machine-learning models in a laboratory-scale evaluation. Guo (33) also reported high technical performance for 3D human body reconstruction. Such results indicate strong technical capability in movement assessment but do not establish improvements in physical activity behavior, exercise adherence, or health outcomes. Chew et al. (32) reported improvements in several behavioral outcomes, including physical activity, in a short-term single-group evaluation. Its seven-day follow-up and lack of a control group limit attribution of these changes to the AI component and provide little basis for judging their sustainability. Algorithmic accuracy and effectiveness at the user or public health level should be treated as distinct outcomes when interpreting evidence in this category.

Prediction or Risk Stratification involves a different use of AI, with clinical, physical, and behavioral variables combined to estimate risk or predict post-intervention outcomes without AI directly delivering the interventions (36–40). Distinguishing prediction from causal effect is essential in this context. Meng et al. (39) and Wei et al. (40) developed machine-learning models using data from randomized exercise trials, but AI neither delivered the exercise interventions nor produced the observed changes in frailty or sarcopenia. Its role was to predict post-intervention status. Forte et al. (37) classified obesity risk using physical fitness variables, while Areias et al. (36) predicted the likelihood of clinical response during a digital musculoskeletal program. Hautala et al. (38) examined risk factors associated with health care costs in the context of cardiac rehabilitation. These applications may be useful for targeting interventions and informing resource allocation, but many of the models were evaluated through internal validation or in limited samples. Their performance in routine practice remains uncertain. External validation, assessment of calibration across different populations, and evaluation of whether model-based predictions lead to better intervention decisions or outcomes represent important next steps for this area of research.

Professional Decision Support shifts the focus from prediction itself to how AI-generated information may inform professional judgment. In several studies, decision support appeared as a secondary function of predictive or assessment models (34, 36, 38). Xu et al. (41), in contrast, directly examined AI support for professional exercise prescription. ChatGPT-4.0 received generally higher expert ratings than the Intelligent Health Promotion System (IHPS) for the accuracy, comprehensiveness, and applicability of personalized exercise prescriptions. Safety concerns remained for cases involving functional movement limitations, and both systems had limitations in medication-related guidance and dynamic adaptation. The prescriptions were evaluated by experts and were not implemented with patients; consequently, clinical effectiveness was not established. Xu et al. (41) provides evidence of decision-support capacity but does not establish that AI can function independently as a clinical decision-maker. This distinction is particularly relevant for older adults and individuals with chronic conditions or comorbidities, for whom clinical appropriateness depends on functional capacity, treatment, and individual risk profiles.

System and Environmental Planning extends the scope of AI beyond individual interventions and clinical decision support. Although represented by only a small number of studies, applications in this area included big data analytics, image analysis, and intelligent governance approaches for assessing environments that support physical activity, informing resource planning, and identifying environmental inequalities (42, 43). Shao et al. (43), for example, used AI-assisted street-view image analysis to identify environmental characteristics and spatial inequalities relevant to physical activity. As actual physical activity behavior and health outcomes were not directly measured, the study informs environmental assessment and planning but does not provide direct evidence of behavioral or health effects. Deng et al. (42) proposed an intelligent governance system integrating individual, family, school, and societal factors, but functions such as physical health monitoring, facility optimization, personalized exercise guidance, and decision support remained largely conceptual and were not empirically tested. This category is particularly relevant from a socioecological perspective because it situates physical activity within the environmental and institutional conditions in which behavior occurs, thereby extending the analysis beyond individual choice or motivation (44). Given the small evidence base and early stage of development, system-level applications currently represent an emerging research direction and cannot yet be considered an established area of practice.

Beyond their functional roles, the practical value of AI applications also depends on how users interact with these systems. Davis et al. (22) reported high technical accuracy for a virtual health assistant during structured weekly interactions, but its ability to respond appropriately to spontaneous user input was substantially lower. Engagement with the assistant was not significantly associated with changes in MVPA. In a user-centered study involving low-income English- and Spanish-speaking women, Figueroa et al. (23) identified personalization, digital literacy, linguistic and cultural appropriateness, privacy, flexibility, and family involvement as key design considerations. Chew (48) reached related conclusions in a scoping review of AI-based conversational agents, highlighting personalization, user engagement, and responsiveness to user needs in the development of digital interventions. Gabarron et al. (49) found that acceptability, satisfaction, and usability were among the human factors most frequently assessed in AI-supported digital physical activity solutions; evidence for effects on physical activity behavior remained limited. Technical performance alone provides little indication of whether an application will be adopted across different user groups or support sustained behavioral change. Public health evaluation should consider accessibility, usability, engagement, cultural appropriateness, and digital inequalities alongside algorithmic performance.

Safety, privacy, and professional oversight become increasingly important as AI-supported applications move toward broader implementation. Systems used in exercise and physical activity may process health information, physical performance measures, and, in some cases, continuously collected user data. Figueroa et al. (23) identified privacy, digital literacy, and linguistic and cultural appropriateness as important user considerations during design. Dergaa et al. (6) raised a related concern in health-specific exercise prescription, where safety must be balanced against adequate personalization and professional evaluation. Wider implementation requires attention to data protection, suitability for different user groups, and professional oversight when recommendations have health implications. Algorithmic bias, transparency, and accountability raise additional concerns. Models developed or validated in narrowly defined populations may perform differently when applied to groups that were underrepresented in the original data. However, these issues were not systematically examined in the studies included in this review and cannot be evaluated in depth from the available evidence.

The distinction between technological capability and practical benefit becomes especially clear across the evidence as a whole. High accuracy in exercise recognition (34, 35), prediction of post-intervention outcomes (39, 40), and generation of personalized exercise intensity or programs (30, 31) demonstrate what AI systems can technically accomplish. Whether these capabilities lead to better health outcomes, sustained physical activity, or more effective service delivery remains a separate empirical question. Favorable outcomes have been reported in some pilot or multicomponent studies (21, 24, 25), but stronger comparative designs have not consistently demonstrated an added benefit of AI-based personalization for primary outcomes (28, 29). The current literature supports neither a general conclusion that AI is ineffective nor that it is consistently superior to existing approaches. Its value appears to vary according to the function being performed, the population, the comparator, and the stage of evidence development. Future research should move beyond developing new systems toward adequately powered controlled studies that can isolate the added value of the AI component, longer-term follow-up, independent external validation, and real-world evaluation across diverse sociodemographic and clinical populations.

### Strengths and limitations

A key strength of this scoping review is the classification of AI-supported exercise and physical activity applications according to their functional roles and stages of evidence development, extending the analysis beyond intervention outcomes alone. This approach provided a common structure for mapping studies that differed substantially in methodology and application context while separating technical development from evaluations of intervention effectiveness. The use of three databases, independent study selection by two reviewers, and reporting in accordance with PRISMA-ScR further enhanced the transparency of the review process.

Several limitations should be considered when interpreting the findings. The review was not prospectively registered, which limits the availability of a time-stamped record of the review methods specified before study selection and data charting. The search was restricted to Web of Science, Scopus, and PubMed, so relevant studies indexed in other databases may not have been identified. Restricting eligibility to English-language articles with accessible full text may have introduced language and accessibility-related selection bias. The search terms were designed to identify studies explicitly linking AI with exercise, physical activity, and public health; relevant research using different terminology may therefore have been missed. Considerable heterogeneity in study designs, populations, AI technologies, and outcomes limited direct comparisons across studies and precluded broad conclusions about the effectiveness of AI applications. A substantial portion of the evidence came from technical development, internal validation, feasibility testing, or small-scale pilot studies, limiting generalizability to other populations and real-world settings.

## Conclusion

AI-supported exercise and physical activity applications span several functions, including intervention delivery or adaptation, monitoring or assessment, risk prediction, professional decision support, and system and environmental planning. From a socioecological perspective, current applications range from individual-level personalization and decision-making within health services to less frequently studied environmental and system-level uses. The breadth of these functions does not indicate equal maturity across the research base. Technical validation and model-development studies have reported promising performance, whereas intervention findings vary from favorable outcomes to no significant added benefit over active comparators or usual care. In multicomponent interventions, the specific contribution of AI often cannot be isolated.

Current evidence suggests that AI may have a complementary role in personalization, monitoring, risk assessment, and professional decision-making; its use as an autonomous substitute for health professionals is not supported by the available evidence. A key question for public health research is which AI functions provide safe, accessible, and measurable added value over existing approaches, for which populations, and under what conditions. Clinical appropriateness and professional oversight need to be considered alongside data security, transparency, algorithmic bias, accountability, and generalizability across populations. Longer-term controlled studies, independent external validation, and real-world evaluations are needed to determine whether technical capabilities translate into sustained physical activity and meaningful public health benefits.

## Data Availability

The original contributions presented in the study are included in the article/supplementary material, further inquiries can be directed to the corresponding author.
